# The ubiquitous and ancient ER membrane protein complex (EMC): tether or not?

**DOI:** 10.12688/f1000research.6944.2

**Published:** 2015-10-05

**Authors:** Jeremy G. Wideman

**Affiliations:** 1 Department of Biosciences, University of Exeter, Exeter, EX4 4QD, UK

**Keywords:** Evolutionary cell biology, ER membrane protein complex (EMC), Membrane contact sites (MCS), ERMES, ER-mitochondria contact sites

## Abstract

The recently discovered endoplasmic reticulum (ER) membrane protein complex (EMC) has been implicated in ER-associated degradation (ERAD), lipid transport and tethering between the ER and mitochondrial outer membranes, and assembly of multipass ER-membrane proteins. The EMC has been studied in both animals and fungi but its presence outside the Opisthokont clade (animals + fungi + related protists) has not been demonstrated. Here, using homology-searching algorithms, I show that the EMC is truly an ancient and conserved protein complex, present in every major eukaryotic lineage. Very few organisms have completely lost the EMC, and most, even over 2 billion years of eukaryote evolution, have retained a majority of the complex members. I identify Sop4 and YDR056C in *Saccharomyces cerevisiae* as Emc7 and Emc10, respectively, subunits previously thought to be specific to animals. This study demonstrates that the EMC was present in the last eukaryote common ancestor (LECA) and is an extremely important component of eukaryotic cells even though its primary function remains elusive.

## Introduction

Recent studies suggest that the EMC (Endoplasmic Reticulum Membrane Complex) is a multifunctional, multi-subunit protein complex. In *Homo sapiens*, the EMC comprises ten subunits, Emc1-10, whereas in *Saccharomyces cerevisiae* the complex comprises only Emc1-6 ( Jonikas *et al.*, 2009). The EMC has been implicated in several cellular processes. It has been implicated in ERAD (ER-associated degradation) ( Christianson *et al.*, 2012; Jonikas *et al.*, 2009; Richard *et al.*, 2013) but the molecular mechanism for how EMC triggers ERAD has remained elusive. Emc6 contains a Rab5 interacting domain and has been shown to interact with Rab5A in humans during autophagosome formation ( Li *et al.*, 2013). It has also been shown that the EMC is an ER-mitochondria tether in *S. cerevisiae* that interacts with the outer membrane protein Tom5 of the TOM (Translocase of the Mitochondrial Outer Membrane) complex ( Lahiri *et al.*, 2014). Most recently, the EMC has been implicated in the proper assembly of multi-pass transmembrane (TM) proteins ( Satoh *et al.*, 2015). These recent findings suggest that EMC involvement in ERAD may be due to secondary effects, as cells devoid of EMC components may result in either disruption of ER-mitochondria tethering, or the misfolding of multipass membrane proteins. Thus, the primary function of the EMC is still open for debate.

The ER-mitochondria encounter structure (ERMES), also involved in ER-mitochondria tethering, is a multifunctional protein complex implicated in both lipid transfer and mitochondrial outer membrane protein assembly ( AhYoung *et al.*, 2015; Kornmann *et al.*, 2009; Meisinger *et al.*, 2006, Meisinger *et al.*, 2007; Wideman *et al.*, 2013; Wideman *et al.*, 2010). However, ERMES as an ER-mitochondria tether is limited to a subset of eukaryote taxa ( Wideman *et al.*, 2013), suggesting that a universal ER-mitochondria tethering complex might exist. Lahiri *et al.* (2014) state in their title that the EMC is a conserved protein complex. However, by stating that a protein is conserved, cell biologists and biochemists often simply mean that the protein is present in *S. cerevisiae* (fungi) and animals. Since the clade comprising animals and fungi only accounts for one fifth of the diversity of eukaryotes ( Adl *et al.*, 2012), more work is necessary in order to support the claim made by Lahiri *et al.* Thus, I was prompted to investigate the taxonomic distribution of the EMC in order to (1) determine if it *really* is a conserved protein complex and (2) if it could possibly represent the pan-eukaryotic ER-mitochondria tether.

## Methods

Sequences of experimentally validated EMC components (see Table S1 for accession numbers) from *H. sapiens* and *S. cerevisiae* were used as queries in BLAST ( Altschul *et al.*, 1997) and pHMMer ( Finn *et al.*, 2011) searches into the predicted proteomes of 70 organisms spanning the diversity of eukaryotes. Retrieved sequences were considered orthologous if they retrieved the original *H. sapiens* or *S. cerevisiae* EMC sequences as top hits when used as reciprocal BLAST or pHMMer queries into *H. sapiens* or *S. cerevisiae* predicted proteomes and did not retrieve any other closely related sequences (except in the case of Emc8 and Emc9, see below). In cases in which EMC components could not be identified in this manner, transcriptomes and genomes were searched using bioinformatically validated sequences from the previous step that were retrieved from closely related species. Genomes were downloaded from public repositories and genome project websites. See Table S1 for retrieved sequences.

## Results and discussion

### The EMC is an ancient and highly conserved protein complex

**Figure 1.**
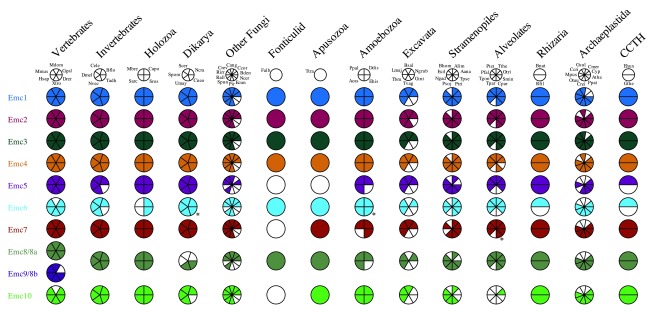
Coulson plot showing distribution of EMC components across eukaryotes. Coloured pies indicate presence of a particular subunit. Plot was generated using the Coulson plot generator ( Field *et al.*, 2013). Asterisks indicate presence of orthologue in a different member of the genus but absent in the indicated species (see Table S1). Abbreviations: Vertebrates: Hsap, *Homo sapiens*; Mdom, *Monodelphis domesticus*; Drer, *Danio rerio*; Xtro, *Xenopus tropicalis*; Ggal, *Gallus gallus*; Mmus, *Mus musculus*; Invertebrates: Cele, *Caenorhabditis elegans*; Dmel, *Drosophila melanogaster*; Bflo, *Branchiostoma floridae*; Nvec, *Nematostella vectensis*; Tadh, *Trichoplax adhaerens;* Unicellular Holozoa: Mbre, *Monosiga brevicollis*; Cowc, *Capsaspora owczarzaki*; Sarc, *Sphaeroforma arctica*; Sros, *Salpingoeca rosetta*; Fungi: Spom, *Schizosaccharomyces pombe*; Scer, *Saccharomyces cerevisiae*; Ncra, *Neurospora crassa*; Cneo, *Cryptococcus neoformans*; Umay, *Ustilago maydis*; Bden, *Batrachochytrium dendrobatidis*; Ncer, *Nosema ceranae*; Ecun, *Encephalitozoon cuniculi*; Pir, *Piromyces sp.*; Spun, *Spizellomyces punctatus*; Rirr, *Rhizophagus irregularis*; Crev, *Coemansia reversa*; Ccor, *Conidiobolus coronatus*; Cang, *Catenaria anguillulae*; Rall, *Rozella allomyces*; Apusozoa: Ttra, *Thecamonas trahens*; Fonticulids: *Fonticula alba*; Amoebozoa: Acas, *Acanthamoeba castellanii*; Ddis, *Dictyostelium discoideum*; Ehis, *Entamoeba histolytica*; Ppal, *Polysphondylium pallidum*; Excavata: Ngru, *Naegleria gruberi*; Gint, *Giardia intesinalis*; Tvag, *Trichomonas vaginalis*; Bsal, *Bodo saltans*; Lmaj, *Leishmania major*; Tbru, *Trypanosoma brucei*; Stramenopiles: Bhom, *Blastocystis hominis*; Alim, *Aurantiochytrium limacinum*; Aana, *Aureococcus anophagefferens*; Tpse, *Thalassiosira pseudonana*; Ptri, *Phaeodactylum tricornutum*; Psoj, *Phytophthora sojae*; Esil, *Ectocarpus siliculosus*; Ngad, *Nannochloropsis gaditana*; Alveolates: Ptet, *Paramecium tetraurelia*; Tthe, *Tetrahymena thermophila*; Otri, *Oxytricha trifallax*; Tpar, *Theileria parva*; Smin, *Symbiodinium minutum*; Tgon, *Toxoplasma gondii*; Cpar, *Cryptosporidium parvum*; Pfal, *Plasmodium falciparum*; Rhizaria: Bnat, *Bigelowiella natans*; Rfil, *Reticulomyxa filosa*; Archaeplastida: Crei, *Chlamydomonas reinhardtii*; Cmer, *Cyanidioschyzon merolae*; Cyp, *Cyanophora paradoxa*; Atha, *Arabidopsis thaliana*; Ppat, *Physcomitrella patens*; Otau, *Ostreococcus tauri*; Gsul, *Galdieria sulphuraria*; Mpus, *Micromonas pusilla*; Ccri, *Chondrus crispus*; CCTH: Ehux, *Emiliania huxleyi*; Gthe, *Guillardia theta*.

**Supplementary Figure S1.**
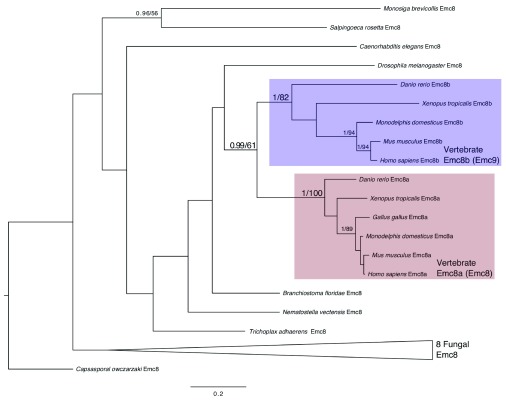
Phylogenetic analysis of opisthokont Emc8/9 proteins. Proteins were aligned using MUSCLE ( Edgar, 2004) and manually adjusted as needed using Mesquite ( http://mesquiteproject.org). Phylogenetic tree reconstructions were carried out using MrBayes v3.2.2 ( Ronquist *et al.*, 2003) for Bayesian analysis. Maximum likelihood bootstrap values were obtained using RaxML ( Stamatakis, 2006) with 100 pseudoreplicates using the LG model ( Le & Gascuel, 2008). Support values: MrBayes/RAxML. Only support values >0.90/50 are shown.

Using homology-searching algorithms EMC candidate proteins were identified in the vast majority of sequenced genomes representing the complete diversity of eukaryotes ( Figure 1). Emc8 and Emc9 are homologues but only a single homologue could be detected in most genomes. A subset of opisthokont Emc8/9 sequences were subjected to a phylogenetic analysis demonstrating that vertebrate Emc8 and Emc9 are the result of a vertebrate-specific duplication of Emc8 ( Figure S1; see legend for methods). Based on this knowledge, I suggest that the vertebrate Emc8 and Emc9 be renamed Emc8a and Emc8b, respectively.

A complete EMC (Emc1-8, 10) was found in at least one representative from each major lineage including animals, fungi, excavates, amoebozoa, green algae, plants, stramenopiles, alveolates, rhizaria, and haptophytes ( Figure 1). The relative sequence conservation of EMC components across diverse taxa suggests that the EMC has an ancient and critical role in cellular function.

### Yeast Sop4 and YDR056C are Emc7 and Emc10, respectively

Although previous reports suggest *S. cerevisiae* EMC comprises only six subunits, I identified Sop4 and YDR056C as orthologues of Emc7 and Emc10, respectively. Supporting this, Jonikas *et al.* (2009), the original discoverers of the EMC, show by co-immunoprecipitation analyses that Sop4 and YDR056C are interacting partners of FLAG-tagged Emc3. This experiment not only confirms my bioinformatic classification but also puts into perspective a previous study on Sop4’s role in membrane protein quality control ( Luo *et al.*, 2002). Furthermore, tracing the evolutionary history of the EMC in fungi demonstrates that Emc8 was lost only in Ascomycetes and a few basally diverging fungi whereas most fungi retain Emc8 (as well as Emc7 and 10).

### The EMC has been independently lost in several lineages

**Figure 2.**
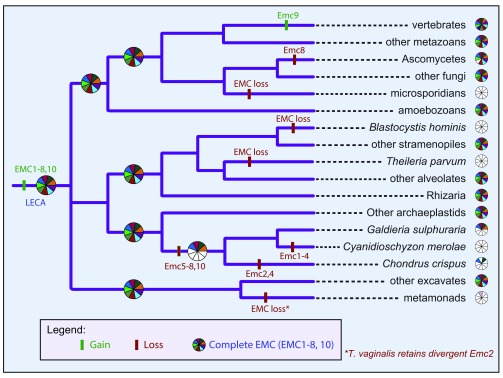
Evolutionary history of the EMC. EMC 1-8 and 10 evolved prior to the divergence of the major eukaryote lineages. Green and red dashes represent gains and losses of EMC components, respectively. Coloured pies are schematic representations of which EMC components were present at different points over the course of evolution.

Although the EMC was identified in representative taxa from every major eukaryote supergroup, I was unable to identify even a single EMC member in the genomes of the microsporidians *Nosema ceranae* and *Encephalitozoon cuniculi*, the metamonad *Giardia intestinalis*, the stramenopile *Blastocystis hominis*, the alveolate *Theileria parva*, and the red alga *Cyanidioschyzon merolae* ( Figure 1 and Figure 2). *Trichomonas vaginalis*, another metamonad retains only a rather divergent Emc2, that passed the test for orthology, but only weakly, suggesting that this protein is under relaxed selection, perhaps repurposed, or in the process of being lost. All other genomes from the remaining 65 species investigated contained clear representatives of EMC homologues ( Figure 1).

These disparate organisms that lack the EMC prompted the question: What cellular or biochemical features tie these diverse organisms together? The microsporidians, metamonads and *B. hominis* all contain reduced anaerobic mitochondria-related organelles (MROs) and also lack the EMC. However, the amoebozoan *Entamoeba histolytica* retains Emc1-4, 7 and 10, the apicomplexan *Cryptosporidium parvum* retains Emc1-4, and 8, and the fungus *Piromyces sp*. retains Emc1-4, 6, 7, and 10, but all three organisms also contain extremely reduced MROs. *T. parva* and *C. merolae* contain relatively normal mitochondria but completely lack the EMC. Thus, it seems that further insight into the cell biology of these organisms is required to understand why only these few species from unrelated lineages have lost the EMC. At this point, of the proposed functions of the EMC, its involvement in multipass membrane protein assembly is the best candidate for generalization to other eukaryotes. It explains the connection to ERAD as a secondary effect of misassembled multipass proteins and explains why an organism with extremely reduced mitochondria ( *E. histolytica*) might retain the EMC. Finally, although EMC involvement as an ER-mitochondria tether is attractive, the distribution of the only known MOM-localized interactor of EMC (Tom5) has not been identified in organisms other than animals and fungi ( Maćasev *et al.*, 2004). Thus, until an ancient interaction partner is identified, the role of EMC as an ancient tether remains speculative.

## Conclusions

Since the vast majority of species from each major branch of eukaryotes retain the EMC it can be inferred that it was present in the last eukaryote common ancestor (LECA). Since the sequences of most of the identified EMC homologues are very similar, it can be inferred that its function has likely been retained in most eukaryote lineages. Thus, the EMC is a generalizable eukaryotic feature as is its function—whatever it might be.

## Data availability

The data referenced by this article are under copyright with the following copyright statement: Copyright: © 2015 Wideman JG

All sequence data are freely available in online databases (NCBI, JGI, or independent genome sequencing project websites).

## Supplementary materials

**Supplementary Table S1**

Protein sequences retrieved in this study.
