# Supplementary material for: The ubiquitous and ancient ER membrane protein complex (EMC): tether or not?
Source: F1000Res. 2015 Oct 5;4:624. Originally published 2015 Aug 25. [Version 2] doi: 10.12688/f1000research.6944.2 (PMC4602282; doi:10.12688/f1000research.6944.2)
Supplement: Peer review history

# Peer review: Referee response for version 2

**DOI:** 10.5256/f1000research.7713.r10104

**Published:** 2015-10-09

**Authors:** Christopher Loewen

**Affiliations:** 1 Department of Cellular and Physiological Sciences, Life Sciences Institute, University of British Columbia, Vancouver, BC, Canada

The article entitled “The ubiquitous and ancient ER membrane protein complex (EMC): tether or not?” authored by Jeremy Wideman presents a solid bioinformatics argument that the EMC protein complex is highly conserved amongst eukaryotes. The EMC was originally identified in budding yeast as a 6 subunit complex (Emc1-6) with a role in protein folding in the endoplasmic reticulum ( Jonikas *et al*., 2009). It was later expanded to 10 subunits  (Emc1-10) in mammals based on proteomic work studying ER-associated degradation ( Christianson *et al., *2011). This current bioinformatics study makes an nice contribution by showing that the whole complex (all ten subunits, with a few exceptions) is widely present in eukaryotes (including invertebrates, fungi and plants). Although overlooked in the original submission, the issue raised by one reviewer about Emc8 and Emc9 being paralogs has now been resolved by the author. 

My main issue with the article is the conclusion drawn by the author that the tethering function of the EMC (discovered by Lahiri *et al*., 2014) is likely not its conserved function. This is arrived at by comparing EMC distribution among species with the presence of mitochondria/MROs. The article title also implies that the findings in this report call into question the role for the EMC in ER-mitochondrial tethering and PS transport (as its conserved function). I feel these are overstatements given the current analysis presented in the paper. Lahiri *et al.*, show interactions between the EMC and Tom5, although these are not demonstrated to be direct, and also state in their discussion that the EMC likely interacts with multiple subunits of the TOM complex (and cite unpublished data to the effect). Hence, judging a role for the EMC in tethering solely based on the presence Tom5 in species seems hardly sufficient to make such an argument. Lahiri *et al.*, demonstrate a role for tethering by the EMC in PS transport to mitochondria, the location of the phosphatidylserine decarboxylase (PSD) that converts the PS into PE. Hence, this transport step is required for PE synthesis by the PSD. Perhaps the author should investigate the coincidence of the EMC and mitochondrial-localized PSD enzymes in the species for which he uses to build arguments against a role for the EMC in tethering. A quick search revealed that two species mentioned in the paper, *C. merolae* and *T. parva*, which completely lack the EMC, contain PSD enzymes that lack mitochondrial-targeting signals (28% and 50% probability, respectively; compared to 95% for *S. cerevisiae* PSD); hence, the EMC would not be needed in these organisms for PE synthesis. The third EMC-lacking species mentioned, *B. hominis*, has a mitochondrial-targeted PSD (99.9% probability) and a second non-mitochondrial PSD (0.01% probability), indicating that there is not an absolute requirement for ER-mitochondrial PS transport and hence, for the EMC in PE synthesis in this species. 

If the author feels that an analysis of the co-occurrence of the EMC and mitochondrial-localized PSD enzymes (and/or TOM complex - all subunits) is beyond the scope of this paper, I feel the paper should be revised, including the title, to de-emphasize the argument that tethering is not a conserved function of the EMC. An additional minor point, the author should name Emc7 and Emc10 on the SGD website for the benefit of the yeast bioinformatic community.

I have read this submission. I believe that I have an appropriate level of expertise to confirm that it is of an acceptable scientific standard, however I have significant reservations, as outlined above.

# Peer review: Referee response for version 2

**DOI:** 10.5256/f1000research.7713.r10674

**Published:** 2015-10-06

**Authors:** Courtney Stairs

**Affiliations:** 1 Centre for Comparative Genomics and Evolutionary Bioinformatics, Department of Biochemistry and Molecular Biology, Dalhousie University, Halifax, NS, Canada

Thank you for addressing my concerns and including a phylogenetic analysis. I think that renaming EMC8 is an excellent way to avoid confusion. I look forward to reading future studies on the topic.

I have read this submission. I believe that I have an appropriate level of expertise to confirm that it is of an acceptable scientific standard.

# Peer review: Referee response for version 2

**DOI:** 10.5256/f1000research.7713.r10675

**Published:** 2015-10-06

**Authors:** Sujoy Lahiri

**Affiliations:** 1 Department of Pharmacology, University of Virginia, Charlottesville, VA, USA

It was gratifying to see that the author has further expanded his study based on my observations. The new finding of Wideman that Emc8 and Emc9 are vertebrate-specific paralogues explains the gain of EMC components in vertebrates. We’ll, however, have to wait for future research to know whether the duplication of Emc8 in these higher eukaryotes has any functional relevance. I feel that the author has rightfully addressed my major concern and thus approve the indexation of this revised manuscript.

I have read this submission. I believe that I have an appropriate level of expertise to confirm that it is of an acceptable scientific standard.

# Peer review: Referee response for version 1

**DOI:** 10.5256/f1000research.7479.r10381

**Published:** 2015-09-24

**Authors:** Courtney Stairs

**Affiliations:** 1 Centre for Comparative Genomics and Evolutionary Bioinformatics, Department of Biochemistry and Molecular Biology, Dalhousie University, Halifax, NS, Canada

The article "The ubiquitous and ancient ER membrane protein complex (EMC): tether or not?" presents the distribution of EMC components across eukaryotic diversity.  Using a strictly bioinformatic approach, Wideman identified homologues of the majority of the EMC in every major eukaryotic supergroup, suggesting that this complex was likely present in the last eukaryotic common ancestor. One of the most interesting findings of this study was the identification of two previously unreported EMC components (Emc7 and Emc10) in yeast.  In fact, the *in silico* findings presented here are supported by previously published co-immunopreciptation study (Jonikas *et al*, 2009) that identified these two components. Surprisingly, the EMC also seems to be present in some organisms that possess highly reduced mitochondria (i.e. mitochondrion-related organelles; MROs). Although beyond the immediate scope of this study, it would be interesting to correlate the presence of various TOM components in these 'amitochondriates' with the various EMC components.  Perhaps a brief comment on this in the discussion would be informative - especially since the interaction of TOM and EMC is known in yeast.  

In another review for this article, Sujoy Lahiri commented on the assignment of the Drosophila and Anopheles Emc8 as Emc9 on the Homologene database (NCBI).  It appears as though these organisms have only one homologue of Emc8 (OR Emc9) by BLAST.  It would be helpful if the author could comment on this observation - is this a mistake by Homologene? A phylogenetic analysis of these two related proteins could be helpful to determine the evolutionary origins of these proteins in animals. They also brought up concerns about the homology between these two proteins - however I think the author addresses this in the methods section where he states '...Emc8 and Emc9, which are related)...'.  

A system so fundamental to the cellular biology of eukaryotes is likely the result of vertical inheritance, however phylogenetic analysis of each component could help solidify this hypothesis and exclude any concerns over lateral gene transfer.  Also, a single sentence describing if any of these components have distant homologues in prokaryotes (especially the recently described Lokiarchaeota) could also be informative for a non-expert audience (such as this reviewer).  

The data presented by Wideman (2015) is well within the scope of F1000Research and will be an invaluable resource for those studying the interactions between the ER and the mitochondria.  I have no major concerns on the article and fully support its continued publication in F1000Research.

I have read this submission. I believe that I have an appropriate level of expertise to confirm that it is of an acceptable scientific standard.

# Response

**Published:** 2015-09-26

**Authors:** Jeremy Wideman

**Affiliations:** University of Exeter, UK

Thank you for your insightful review. I have addressed the major concern from Sujoy Lahiri's review by including additional data (opisthokont Emc8/9 phylogeny).

As you suspected, some of your other comments are out of the scope of the paper, but I would like to comment on them here for anyone that is interested.

First, regarding your comment “it would be interesting to correlate the presence of various TOM components in these 'amitochondriates' with the various EMC components”: yes this would be interesting, however, I believe best included in a larger study on the evolution of protein import pathways. Tom5, the only known MOM interactor of EMC is found only in Opisthokonts (Macasev et al. 2004), although this has not been investigated in detail for quite some time. The protein is so short (~50aa) that it is easily missed in bioinformatic analyses; even if the protein is more widespread, it may be that it will only be identified biochemically. Additionally, most amitochondriates have extremely divergent Tom complexes (e.g. *Entamoeba*, microsporidians, *Giardia*), and it is unlikely that even if a small protein like Tom5 is present in these organisms that it will be detectable by phylogenetic analysis.

Second, prokaryotes do not seem to have any close homologues (based on a preliminary BLAST into NCBI prokaryote database) but some weak homology can be detected. Further investigation is beyond the scope of this project.

Third, the likelihood of HGT of EMC components is quite low in this case given the high frequency of retention of EMC across all eukaryotes. Also, for many of the proteins it is unlikely that phylogenies would resolve HGTs as many of the proteins are very short and support values would be low.

# Peer review: Referee response for version 1

**DOI:** 10.5256/f1000research.7479.r10100

**Published:** 2015-09-04

**Authors:** Sujoy Lahiri

**Affiliations:** 1 Department of Pharmacology, University of Virginia, Charlottesville, VA, USA

The article titled “The ubiquitous and ancient ER membrane protein complex (EMC): tether or not?” authored by Jeremy G. Wideman is a comprehensive study to determine if the EMC proteins are truly conserved. Using homology searching algorithms the author has shown that except for a few branches the EMC proteins are present in the vast majority of the eukaryotes and reasoned in favor of the presence of EMC proteins in the last eukaryote common ancestor (LECA). In addition the author has also identified two genes in *S. cerevisiae *to be orthologues of Emc7 and Emc10 which supports the finding of Jonikas *et al*. where these two proteins were co-immunoprecipitated along with the other EMC proteins.

In light of the increasing scientific attention on the EMC proteins over past few years and their multifaceted roles in cell physiology I find this article to be quite relevant in delivering a thorough understanding of this protein complex from the evolutionary perspective. Thus this study by Wideman will be helpful in further understanding of the biology of the EMC proteins. On its scientific merit I consider this article to be substantially important for getting published with F1000Research.

However there is one major concern, which I'd like to be addressed before endorsing the acceptance of this article. The author has described Emc9 to be present only among the vertebrates. However the HomoloGene database of NCBI shows Emc9 homologs to be present in *Drosophila melanogaster* and *Anopheles gambiae  *( http://www.ncbi.nlm.nih.gov/homologene/41095). I assume that the homology searching algorithm used by the author has designated the Drosophila Emc9 homolog protein NP_611731.1 as Emc8 which calls for a discussion by the author. Furthermore, this led me to explore whether Emc8 and Emc9 share any sequence homology. Using pairwise alignment of NCBI Blast ( http://goo.gl/B8T0P3) between human Emc8  (NP_006058.1) and Emc9 (NP_057133.2) I found 44% sequence identity between these two proteins with 93% query coverage and an E value of 2e-57. No other Emc proteins, besides Emc8 and Emc9, share such high degree of sequence identity. This makes me curious of whether Emc8 and Emc9 could be paralogs in the vertebrates. In such case the gain of Emc9 among the vertebrates could be explained by a possible duplication of Emc8. In light of this I would request the author to elucidate possible reasons for the high degree of sequence homology between Emc8 and Emc9 and discuss the anomaly between his data as presented in this article and the HomoloGene database.

I have read this submission. I believe that I have an appropriate level of expertise to confirm that it is of an acceptable scientific standard, however I have significant reservations, as outlined above.

# Response

**Published:** 2015-09-26

**Authors:** Jeremy Wideman

**Affiliations:** University of Exeter, UK

Thank you for your very positive review. I have addressed your major concern by including additional phylogenetic data. Emc8 and Emc9 are now clearly shown as paralogues due to a duplication in the ancestral lineage leading to vertebrates. As such, to prevent future confusion I suggest that vertebrate Emc8 and Emc9 be renamed to Emc8a and Emc8b respectively. I hope you now find the article sufficient for approval.
